# Supplementary material for: Transposon insertion sequencing of Pseudomonas aeruginosa identifies multiple intersecting pathways essential for extreme colistin resistance
Source: PLoS Pathog. 2026 Sep 8;22(9):e1014168. doi: 10.1371/journal.ppat.1014168 (PMC13552734; doi:10.1371/journal.ppat.1014168)

For S4 Fig. A

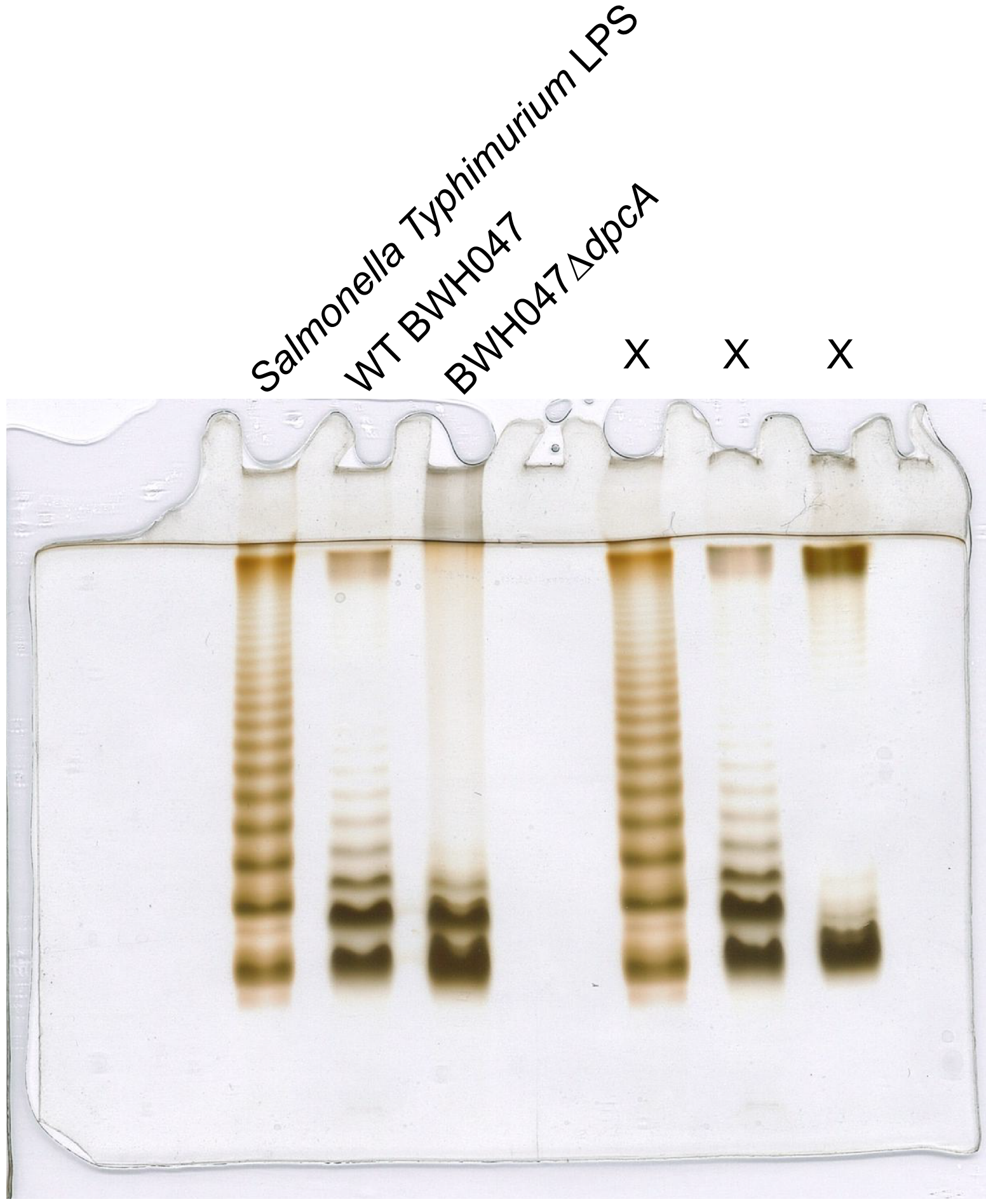

For S4 Fig. B

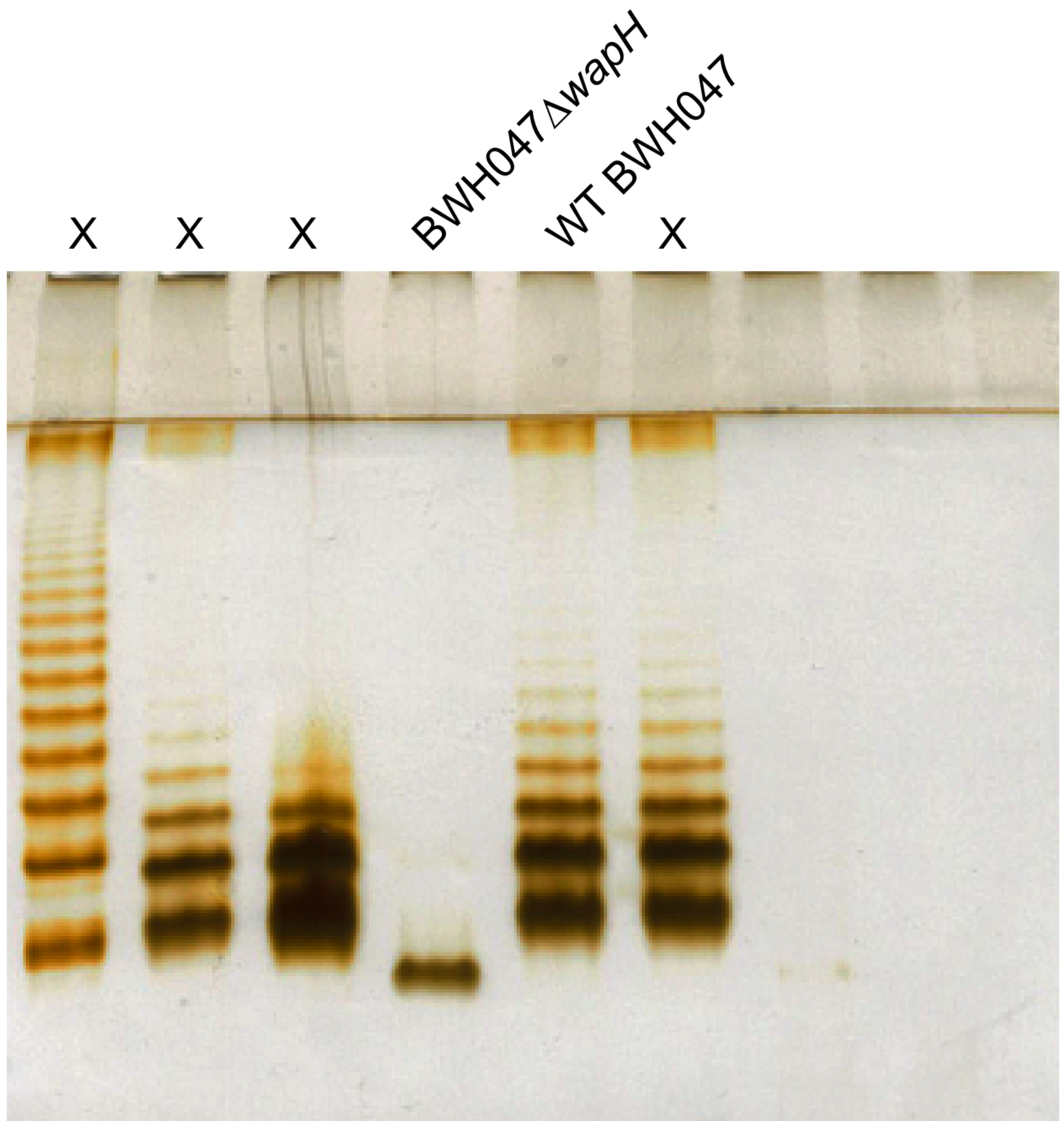

Supplement: Supplementary file 1 [file ppat.1014168.s025.pdf]
